# Supplementary material for: A fluid mechanic’s analysis of the teacup singularity
Source: Proc Math Phys Eng Sci. 2020 Aug 26;476(2240):20200348. doi: 10.1098/rspa.2020.0348 (PMC7482198; doi:10.1098/rspa.2020.0348)
Supplement: Supplemental text [file rspa20200348supp1.pdf]

Electronic Supporting Material:
A fluid mechanic's analysis of the teacup singularity

Dwight Barkley
Mathematics Institute, University of Warwick, Coventry, CV4 7AL
(Dated: July 7, 2020)

I. SADDLE FLOW AND APPROXIMATE SYMMETRY

The meridional velocity field in the vicinity of the critical ring is very nearly, but not exactly, anti-symmetric under reflection $(r, z) \to (1 - z, 1 - r)$, for $z \geq 0$. Figure 1(a) shows a contour plot of the Stokes streamfunction $\psi(r, z)$ in the vicinity of the critical ring. The contour lines are streamlines of the meridional flow. Also shown are contours of the reflected streamfunction $\psi(1 - z, 1 - r)$. The two sets of contours are barely distinguishable, demonstrating just how nearly symmetrical the meridional streamlines are.

Figure 1(b) shows velocity profiles along two cuts indicated by the two lines in Fig. 1(a). The red curve is u_z as a function of r and the blue curve is u_r as a function of z. These are nearly identical except very close to the right side of the plot, which corresponds to the cylinder wall for the red curve and the and midplane for the blue curve. There is shear (vorticity) at the cylinder wall $\partial_r u_z|_{r=1} \neq 0$ but not at the midplane $\partial_z u_r|_{z=0} = 0$.

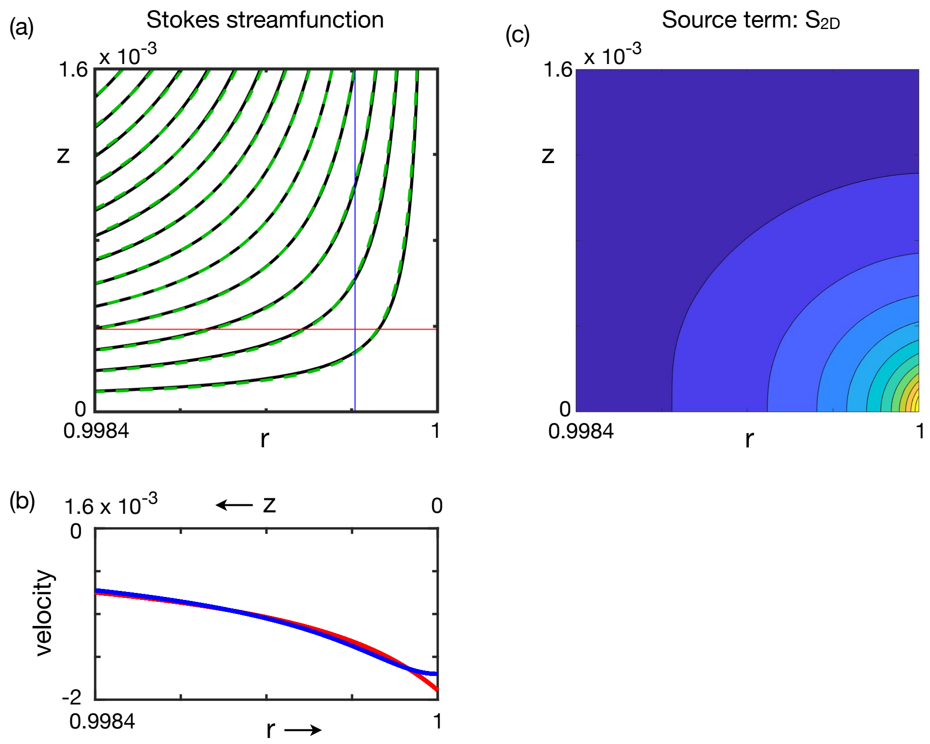

FIG. 1: (a) Approximate symmetry of the meridional flow near the critical ring. Contours of the Stokes streamfunction $\psi(r, z)$ are shown in black. Also shown in dashed green are contours of $\psi(1 - z, 1 - r)$. The two sets of contours are nearly identical. (b) Velocity profiles in along the cuts indicated by red and blue lines in (a). The z coordinate is oriented to align the profiles. The red curve is $u_z(r, z = 3.9 \times 10^{-4})$ while the blue curve is $u_r(r = 1 - 3.9 \times 10^{-4}, z)$. Note that $\partial_r u_z|_{r=1} \neq 0$ so that there is shear (vorticity) at the cylinder wall, $r = 1$. However, by symmetry $\partial_z u_r|_{z=0} = 0$ and there is no shear (vorticity) at the midplane $z = 0$. (c) Plot of the source term S_{2D} for the meridional pressure Poisson equation. The lack of exact symmetry under $(r, z) \to (1 - z, 1 - r)$ is evident.

The streamlines lacks exact symmetry under the transformation $(r, z) \to (1 - z, 1 - r)$ both because the inhomogeneous radial coordinate is not equivalent to the homogeneous axial coordinate and because the symmetry condition at the midplane $z = 0$ does not apply at the cylinder wall $r = 1$. The result in Fig. 1(b) suggest that the second factor is much more relevant than the first in the lack of symmetry in the meridional saddle flow. It further suggests

that this lack of exact symmetry would remain in the Boussinesq system. Only by replacing the symmetry condition at the midplane with a wall in the Boussinesq system could one hope to achieve a symmetric saddle flow.

For completeness, Fig. 1(c) shows the source term S_{2D} for the meridional pressure Poisson equation. The lack of exact symmetry under $(r, z) \to (1-z, 1-r)$ is evident.

II. EVOLUTION TOWARD SINGULARITY

Figure 2(a) shows the time evolution of $\partial_z u_\theta^2$ over just a portion of the cylinder wall. While the final time investigated in the main paper is $t = 0.0031$, the simulations are adequately resolved to $t = 0.0032$ and so this case is also shown here. Recall from Sec. 6(c) of the main paper that P_a is determined from the Hilbert transform of $\partial_z u_\theta^2(z)$, evaluated at zero and that blowup is controlled by

$$-\frac{H(\partial_z u_\theta^2)(0)}{W^2}. \tag{1}$$

We can investigate whether this approaches a finite limit as the flow evolves. Using the integral representation of the Hilbert transform, this can be written as

$$-\frac{H(\partial_z u_\theta^2)(0)}{W^2} = \frac{1}{W^2}\frac{1}{\pi}\int_{-\infty}^{\infty}\frac{\partial_z u_\theta^2(z)}{z}\,dz = \frac{2}{\pi}\int_0^{\infty}\frac{1}{W^2 z}\partial_z u_\theta^2(z)\,dz = \frac{2}{\pi}\int_0^{\infty} h(\xi)\,d\xi,$$

where

$$\xi = \frac{2\Omega^2}{W^2}z, \quad h(\xi) = \frac{1}{W^2\xi}\partial_z u_\theta^2(\frac{W^2}{2\Omega^2}\xi). \tag{2}$$

The coordinate ξ is the unique rescaling of z such that $\lim_{\xi\to 0} h(\xi) = 1$.

Figure 2(b) shows the time evolution of the integrand $h(\xi)$ for the same data as in Fig. 2(a). The curves suggest convergence to a finite limit, thereby implying a finite-time blowup. We already have evidence from Luo & Hou that the flow collapses to a singularity in a nearly, but not exactly, self-similar way [1–4]. Hence this is not a new result, but rather a different way of looking at what is already known. The lack of exact self-similarity necessarily follows since the data is taken from simulations in an axially periodic cylinder and not an infinite cylinder. Therefore the integrands $h(\xi)$ fundamentally cannot collapse because the (very weak) tails at large ξ cannot. This lack of exact self-similarity for this flow is well known [3, 4].

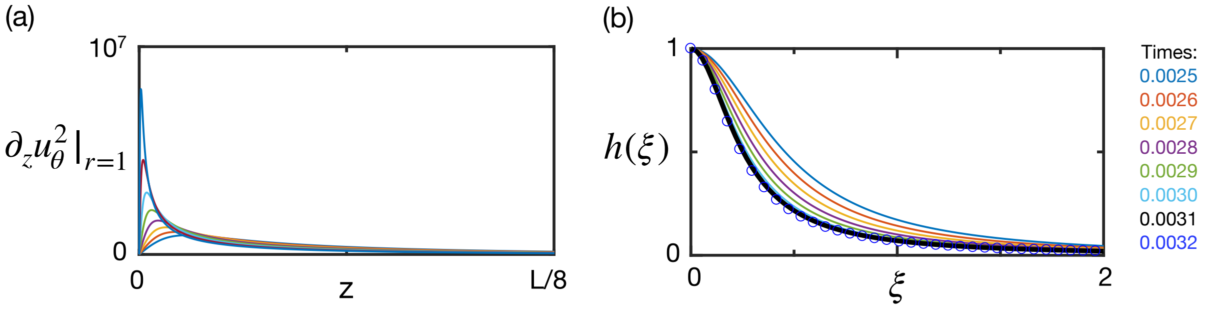

FIG. 2: (a) Time evolution of $\partial_z u_\theta^2$ along the cylinder wall. These profiles determine the pressure curvature of p_a on the critical ring. (b) Plots of h versus ξ given by expressions Eqs. (2) for the same data as in (a). The bold black curve corresponds to the time $t = 0.0031$. Results in the main paper are all shown at this time. For reference, at $t = 0.0031$, $\xi = 2$ corresponds to $z = 1.51 \times 10^{-3}$, which is very close to $\delta = 1.6 \times 10^{-3}$ used for most plots in the main paper. Circles are used to show the last adequately resolved time in the simulations, $t = 0.0032$.

[1] G. Luo and T. Y. Hou, Proc Natl Acad Sci USA **111**, 12968 (2014).
[2] G. Luo and T. Y. Hou, Multiscale Model Sim. **12**, 1722 (2014), ISSN 1540-3459.
[3] D. Chae and T.-P. Tsai, J. Nonlinear Sci. **25**, 193 (2015).
[4] G. Sperone, J. Nonlinear Sci. **27**, 1325 (2017), ISSN 0938-8974.
